# Comprehensive characterization of maternal, fetal, and neonatal microbiomes supports prenatal colonization of the gastrointestinal tract

**DOI:** 10.1038/s41598-023-31049-1

**Published:** 2023-03-21

**Authors:** Jee Yoon Park, Huiyoung Yun, Seung-been Lee, Hyeon Ji Kim, Young Hwa Jung, Chang Won Choi, Jong-Yeon Shin, Joong Shin Park, Jeong-Sun Seo

**Affiliations:** 1 Department of Obstetrics and Gynecology, Seoul National University College of Medicine, Seoul, Republic of Korea; 2 Department of Obstetrics and Gynecology, Seoul National University Bundang Hospital, Gyeonggi-do, Republic of Korea; 3 Department of Pediatrics, Seoul National University College of Medicine, Seoul, Republic of Korea; 4 Department of Pediatrics, Seoul National University Bundang Hospital, Gyeonggi-do, Republic of Korea; 5 Precision Medicine Center, Seoul National University Bundang Hospital, Gyeonggi-do, Republic of Korea; 6 Macrogen Inc, Seoul, Republic of Korea

**Keywords:** Clinical microbiology, Microbial communities, Biological techniques, Genetics, Microbiology, Molecular biology, Medical research, Molecular medicine

## Abstract

In this study, we aimed to comprehensively characterize the microbiomes of various samples from pregnant women and their neonates, and to explore the similarities and associations between mother-neonate pairs, sample collection sites, and obstetrical factors. We collected samples from vaginal discharge and amniotic fluid in pregnant women and umbilical cord blood, gastric liquid, and meconium from neonates. We identified 19,597,239 bacterial sequences from 641 samples of 141 pregnant women and 178 neonates. By applying rigorous filtering criteria to remove contaminants, we found evidence of microbial colonization in traditionally considered sterile intrauterine environments and the fetal gastrointestinal track. The microbiome distribution was strongly grouped by sample collection site, rather than the mother-neonate pairs. The distinct bacterial composition in meconium, the first stool passed by newborns, supports that microbial colonization occurs during normal pregnancy. The microbiome in neonatal gastric liquid was similar, but not identical, to that in maternal amnionic fluid, as expected since fetuses swallow amnionic fluid in utero and their urine returns to the fluid under normal physiological conditions. Establishing a microbiome library from various samples formed only during pregnancy is crucial for understanding human development and identifying microbiome modifications in obstetrical complications.

## Introduction

The human microbiome potentially carries the answer to many secrets of the human body. It has been linked to maintaining homeostasis in health and is associated with numerous diseases^1,2^. Recent research has shifted to explore the microbiome in less-studied populations, such as infants or pregnant women, to better understand its role in human development. Microbiome development is likely to start from the in-utero environment and changes in a lifetime, continuously affecting the immune system and metabolism. Pregnancy has been shown to alter microbial populations within the maternal body and may impact future maternal, fetal, and neonatal health^3^. Pregnancy allows temporary immunotolerance to a foreign body, facilitating microbiome remodelling and potential adaptations to the immune system and metabolism^4^. Some microbiome studies in pregnancy have proposed that fetal environments, including placenta and amniotic fluid, traditionally known as sterile, contain several characteristic microbiotas not identified in routinely performed culture techniques^5,6^. However, the biomass of these microbiotas is small and the reliability of the sequencing methods and potential for contamination have been criticized. The association between those microbiota and specific obstetric conditions has not yet been proven and warrants further investigation.

The vagina is the most commonly studied site of bacteria in the female reproductive organ, as it is connected to the uterus through the cervix and is exposed to the skin. Microbiome research in pregnancy, however, has advanced slowly due to ethical concerns and difficulties in accessing samples. Aagaard et al. found that the vaginal microbiome changes during pregnancy based on gestational age and that *Lactobacillus* species play a role in preventing pathogenic bacterial growth^7^. More specifically, pregnancy leads to decreased diversity, increased proportion of *Lactobacillus*, and higher stability in the vaginal microbiome^8,9^. Some vaginal bacteria have been linked to preterm birth via intrauterine inflammation or infection^10–14^, yet there are no clinical guidelines for testing or monitoring these microbiota. Other sites that had been evaluated for microbiome in pregnancy are maternal^15^, oral cavity^16^, placenta^5^, amniotic fluid^17,18^, and neonatal gut^19^; but previous studies were fragmentary and more systematic research is needed.

In this study, we have comprehensively characterized the microbiome in vaginal discharge (VD) and amniotic fluid (AF) from pregnant women and in umbilical cord blood (CB), gastric liquid (GL), and meconium (M) from their neonates. The goal was to determine the relationships between these samples and various obstetric conditions.

## Results

### Description of the study populations and clinical characteristics

A total of 141 low-risk pregnant women were enrolled sequentially and 178 neonates were born from the study population with 37 cases being twin pregnancies. All women were of Asian ethnicity (Korean), and the median age was 34 (interquartile range 31–37) years (Table 1). The proportion of nulliparity was slightly over half of the population (67%), and the median values of height, weight, and body mass index (BMI) were 162 cm, 70 kg, and 27 kg/m^2^, respectively. About 30% were conceived by assisted reproductive technology (ART), including intrauterine insemination (IUI) and in vitro fertilization with embryo transfer (IVF-ET). As mentioned above, twin pregnancy was approximately one-fourth of the total population, and among them, 19% were monochorionic. The median gestational age at delivery was 37.7 weeks (interquartile range 36.9–38.6), and preterm birth before 37 weeks of gestation was 26.2% (37/141). The rate of cesarean section was 55% (77/141). Seven neonates had congenital structural anomalies (atrioventricular septal defect, absence of corpus callosum in the brain, achondroplasia, cleft lip, polydactyly, and syndactyly), which did not directly affect the neonatal survival. The frequencies of other obstetric complications or underlying maternal diseases are described in Table 1.

**Table 1:** Clinical characteristics of the study population.

Characteristics	Values
Age (years)	34 (31–37)
Nulliparity	67.4% (95/141)
Height (cm)	162.4 (159.5–165.1)
Weight (kg)	69.9 (65.7–77.0)
BMI (kg/m^2^)	27.0 (25.1–29.8)
Pregnancy from IVF-ET	24.1% (34/141)
Pregnancy from IUI	5.0% (7/141)
Twin pregnancy	26.2% (37/141)
Monochorionic twins	18.9% (7/37)
Gestational age at delivery (weeks)	37.7 (36.9–38.6)
Preterm birth before 37 weeks	26.2% (37/141)
Cesarean section	54.6% (77/141)
Epidural anesthesia	47.5% (67/141)
Birthweight (g)^a^	2800 (2480–3124)
Male neonates^a^	50.0% (89/178)
Low Apgar score < 7 in 1 min^a^	3.9% (5/178)
Low Apgar score < 7 in 5 min^a^	0.6% (1/178)
Meconium staining^a^	2.2% (4/178)
Congenital structural anomaly^a^	3.9% (7/178)
Obstetric complications and underlying diseases	
Use of tocolytics due to preterm labor	8.5% (12/141)
Preterm premature rupture of membranes	3.5% (5/141)
Cerclage operation	4.3% (6/141)
Preeclampsia	12.1% (17/141)
Chronic hypertension	2.1% (3/141)
Fetal growth restriction	2.8% (4/141)
Oligohydramnios in the 3rd trimester	5.0% (7/141)
Gestational thrombocytopenia	3.5% (5/141)
Gestational diabetes	13.5% (19/141)
Pregestational diabetes	0.7% (1/141)
Placenta previa	2.1% (3/141)
Placental abruption	0 (0/141)
Myoma uteri on ultrasound	12.1% (17/141)
Endometriosis confirmed before pregnancy	1.4% (2/141)
Hypothyroidism	9.9% (14/141)
Hyperthyroidism	0.7% (1/141)
Allergic diseases^b^	4.3% (6/141)
Psychologic diseases on medication^c^	2.8% (4/141)

BMI, body mass index; IVF-ET, in-vitro fertilization and embryo transfer; IUI, intrauterine insemination.

Values are expressed as the median (interquartile range) for continuous variables and percentage for categorical variables.

^a^The denominator is the number of newborns.

^b^Asthma for three cases, allergic rhinitis, angioedema, and cholinergic urticaria.

^c^Major depressive disorder for two cases, anxiety disorder, and panic disorder.

### Maternal and neonatal microbiome landscape during delivery

**Figure 1:**
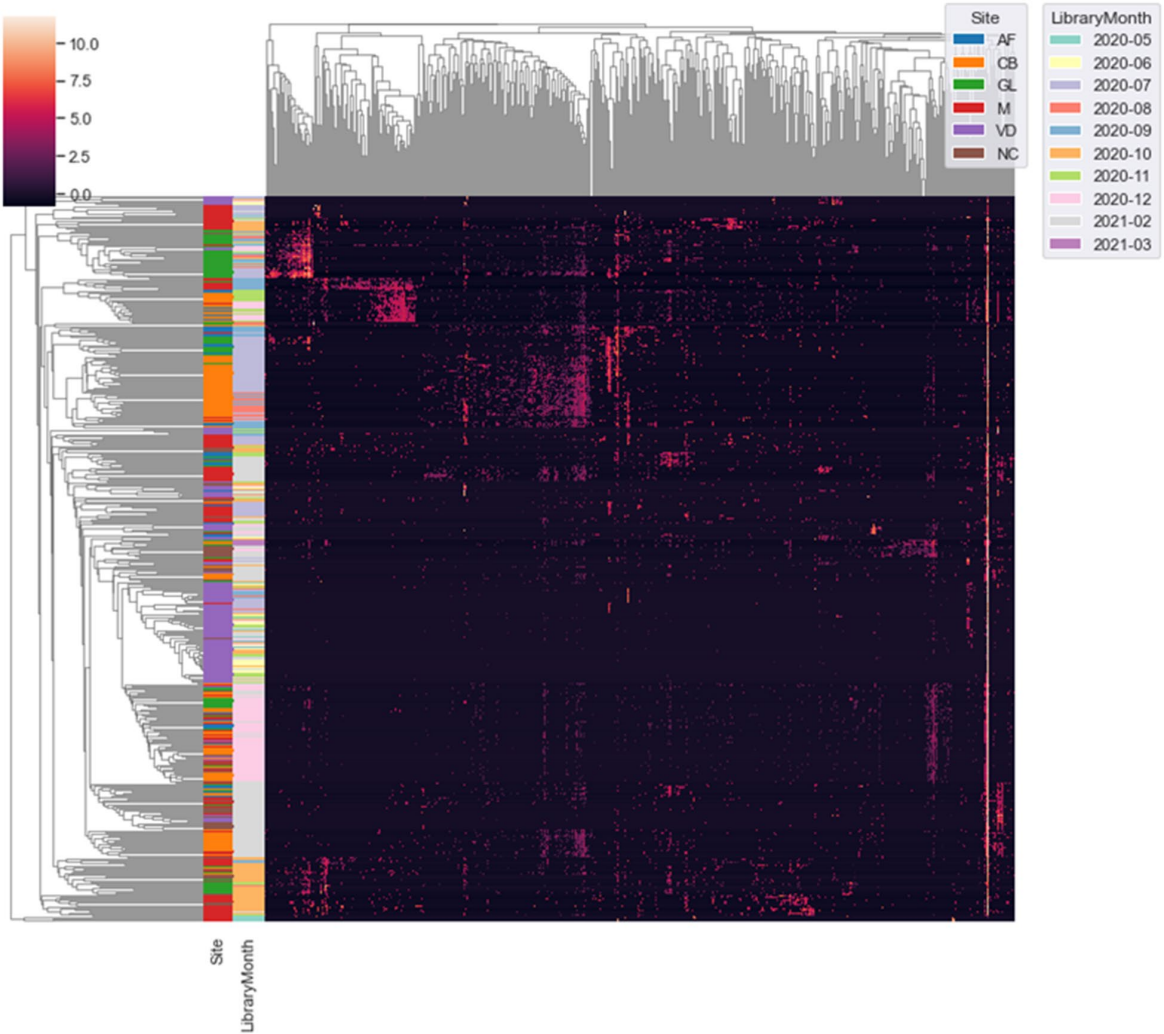
Batch effect detection in 16S rRNA amplicon sequencing data. Center log-ratio transformation was used to normalize the filtered ASV table before generating a hierarchically clustered heatmap based on correlation coefficients. AF, amniotic fluid; CB, umbilical cord blood; GL, gastric liquid; M, meconium; VD, cervicovaginal discharge; NC, negative control.

We identified 19,597,239 bacterial sequences and 22,412 unique amplicon sequence variants (ASVs) from 641 samples, including cervicovaginal discharge (n = 154), amniotic fluid (n = 40), gastric liquid (n = 100), umbilical cord blood (n = 125), meconium (n = 160), and negative controls (n = 62). The ASVs were taxonomically annotated, but we found evidence of batch effects in our sequencing data for all sample types except VD (Fig. 1 and Supplementary Fig. S1). The batch effects were likely introduced during library construction for next-generation sequencing (NGS) and not during sequencing itself (Supplementary Fig. S2). However, this was expected because our samples were collected from body sites with low-biomass specimens, making our samples prone to contamination^20^. Therefore, we expected to find many false positives and applied a series of filters, as outlined in Supplementary Fig. S3. Notably, we found and removed 203 ASVs that were statistically determined as contaminants because they were highly prevalent in negative controls (Supplementary Fig. S4) or they showed higher frequencies in low-concentration samples (Supplementary Figs. S5 and S6).

**Figure 2:**
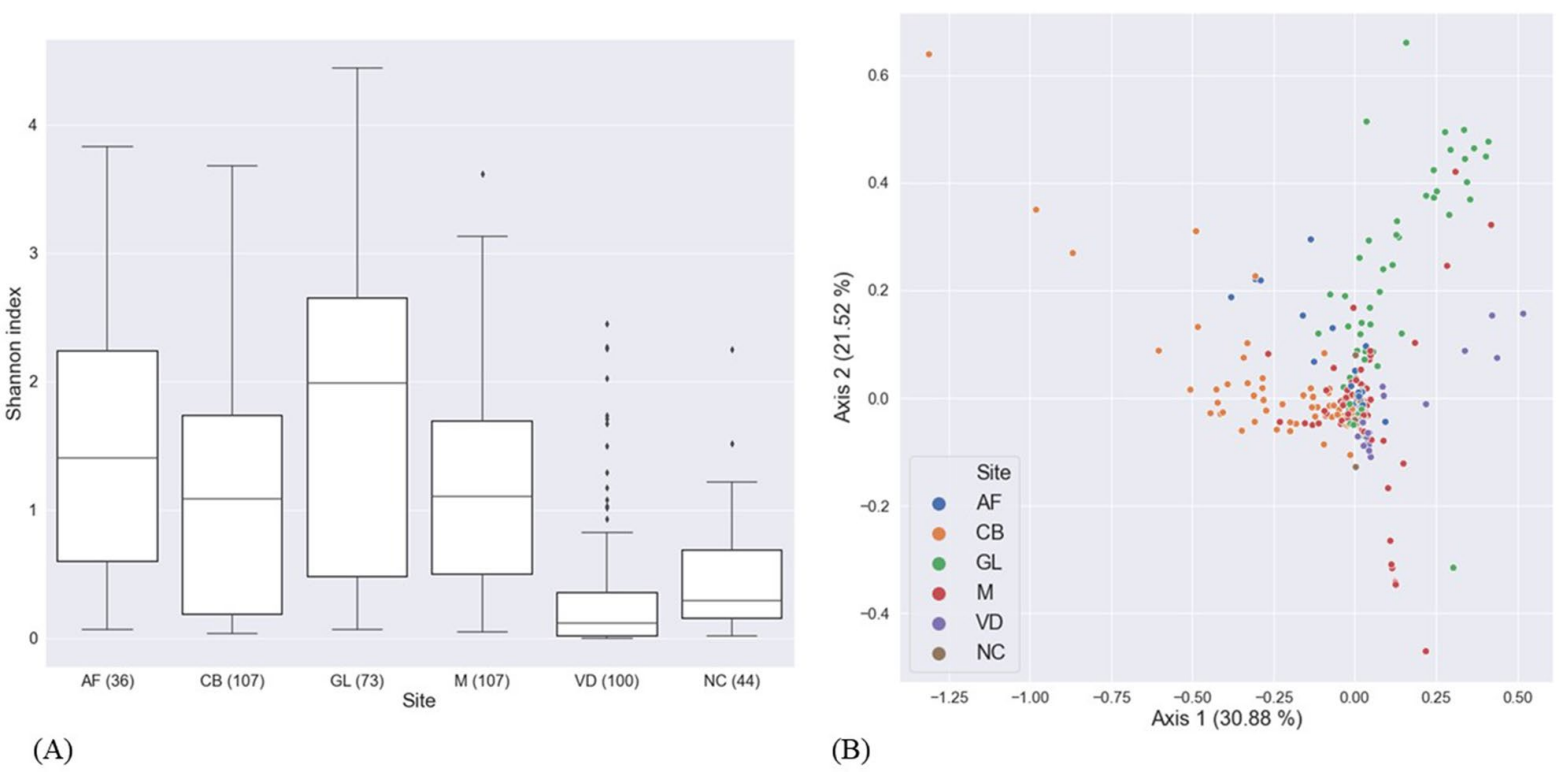
Alpha and beta diversity of the Korean maternal and neonatal microbiome. (**A**) Alpha diversity: The filtered ASV table was rarefied before Shannon index was computed for each sample. The VD group exhibited the least amount of alpha diversity. AF, amniotic fluid; CB, umbilical cord blood; GL, gastric liquid; M, meconium; VD, cervicovaginal discharge; NC, negative control; (**B**) Beta diversity: The filtered ASV table was rarefied before the samples were projected into 2D-space with principal coordinates analysis using the weighted UniFrac distance.

We measured the alpha diversity of the samples by calculating Shannon indices (Fig. 2A). The alpha diversity decreased in the following order: GL, AF, M, CB, and VD. The negative control group showed a slightly higher diversity compared to the VD sample, suggesting that negative controls for 16S amplicon sequencing can have microbiome diversity as rich as real biological specimens. Next, we estimated the beta diversity of our samples by computing the weighted UniFrac distances (Fig. 2B). The samples were moderately well separated by sample collection site when projected using principal coordinates analysis (PCoA).

To determine if mothers share microbes with their newborns, we compared the average number of ASVs shared between mother-neonate pairs and 1000 pairs randomly selected from different families. We conducted a one-tailed t-test and found that mother-neonate pairs did not have a higher number of shared ASVs compared to randomly selected pairs (N = 0.7 vs. N = 1.8, respectively; p = 1.0).

### Clinical relevance of microbiome in pregnancy

To better understand the sources of variation seen in the beta diversity of our samples, we carried out the permutational multivariate analysis of variance (PERMANOVA) using different factors, including clinical information. As shown in Table 2, when all sample types were included in the analysis, the variable “Site” explained 17.2% of the variation (p-value = 0.001), and the variable “LibraryMonth,” was 7.4% (p-value = 0.002). This result indicates that the samples could still be separated well based on the microbiome pattern unique to their body site, despite the significant batch effects present within our dataset. When the analysis was restricted to each sample type, except for the sample VD group, the variable “LibraryMonth” was found to be significant for all sample types. The explanatory power increased to a range between 24.5 and 48.9%. These results align with the hypothesis that our samples are predominantly low-biomass specimens and prone to contamination.

**Table 2:** Summary of the results (R^2^ and p-values) from permutational multivariate analysis of variance (PERMANOVA).

Variable	All sites	AF	CB	GL	M	VD
Site	**0.172 (0.001)**	–	–	–	–	–
LibraryMonth	**0.074 (0.002)**	**0.489 (0.002)**	**0.338 (0.008)**	**0.245 (0.016)**	**0.26 (0.001)**	0.051 (0.834)
Age	0 (0.947)	0.021 (0.506)	0.012 (0.2)	0.019 (0.248)	0.016 (0.151)	0.016 (0.211)
PretermBirth37	0.002 (0.445)	0.029 (0.317)	0.003 (0.826)	0.012 (0.469)	**0.026 (0.017)**	0.004 (0.7)
DeliveryMethod	0.003 (0.206)	0.01 (0.676)	0.011 (0.225)	0.038 (0.069)	0.008 (0.473)	**0.06 (0.005)**
HasGDM	0.002 (0.365)	0.025 (0.372)	0.015 (0.134)	0.012 (0.446)	0.018 (0.086)	0.002 (0.891)
IVFET	0.001 (0.934)	0.01 (0.845)	0.002 (0.934)	0.014 (0.364)	0.004 (0.854)	0 (0.99)
Epidural	0.003 (0.162)	0.03 (0.328)	0.018 (0.108)	0.005 (0.76)	0.006 (0.559)	0.006 (0.47)
InducedLabor	0.002 (0.373)	0.014 (0.676)	0.003 (0.839)	0.02 (0.226)	0.01 (0.378)	0.002 (0.828)
Hypertension	0.009 (0.135)	0.038 (0.573)	0.047 (0.119)	0.011 (0.875)	0.03 (0.363)	0.019 (0.434)
Weight	0.002 (0.483)	0.011 (0.776)	**0.022 (0.039)**	0.003 (0.938)	0.006 (0.59)	0.011 (0.321)
HasTwins	0.001 (0.544)	0.009 (0.802)	0.006 (0.451)	0.016 (0.338)	0.01 (0.369)	0.007 (0.463)
BabySex	0.005 (0.263)	0.024 (0.839)	0.019 (0.261)	0.031 (0.398)	0.015 (0.63)	0.022 (0.387)
AntibioticsUse	0.004 (0.086)	–	0.025 (0.06)	0.007 (0.665)	**0.031 (0.035)**	0.014 (0.23)
Residuals	0.719	0.29	0.478	0.568	0.56	0.786
Total	1	1	1	1	1	1

AF, amniotic fluid; CB, umbilical cord blood; GL, gastric liquid; M, meconium; VD, cervicovaginal discharge.

Significant values are in bold.

**Figure 3:**
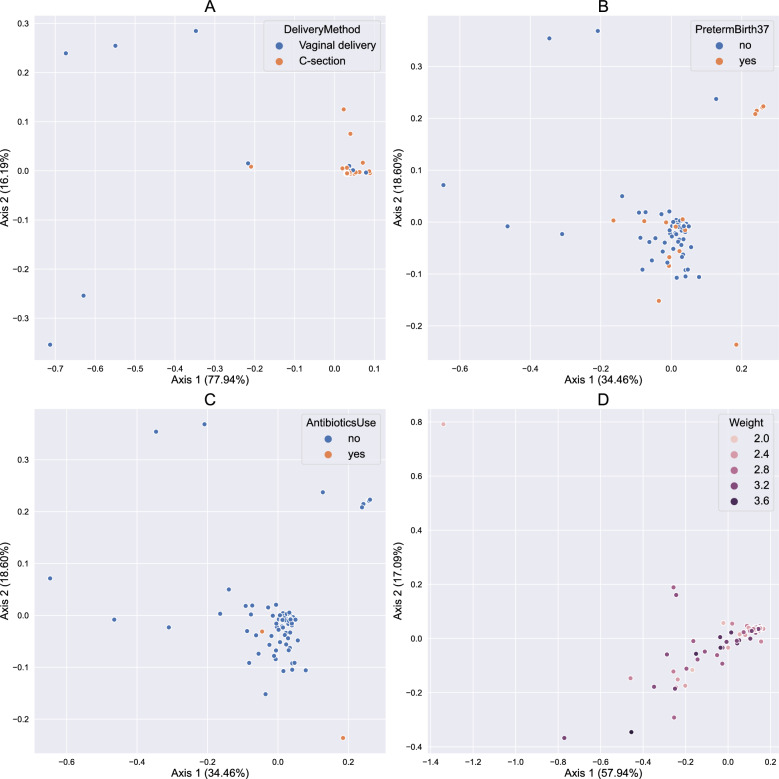
Beta diversity results of the PERMANOVA analysis. Principal coordinates analysis using weighted UniFrac distance is shown for (**A**) the cervicovaginal discharge samples, (**B**) and (**C**) the meconium samples, and (**D**) the umbilical cord blood samples.

Additionally, the variable “DeliveryMethod” was returned as significant for the VD group, the variables “PretermBirth37” and “AntibioticsUse” for the M group, and the variable “Weight” for the CB group (Fig. 3). We explored the significant variables in each group using PCoA with weighted UniFrac distance. Several ASVs of *Lactobacillus* and one ASV of *Gardnerella* were found in the VD group. In the M group *Staphylococcus* showed a strong association with preterm birth. Lastly, the lists of bacterial taxa were connected to the weights of neonates in the CB group. Table 3 shows the analysis of the composition of microbiomes (ANCOM) for various clinical data to study any statistically significant relevance with bacteria in multiple sample types.

**Table 3:** Summary of the results from analysis of composition of microbiomes (ANCOM) at the genus level.

Variable	Site	Taxon	W score	Results
DeliveryMethod	AF	d__Bacteria;p__Firmicutes;c__Clostridia;o__Peptostreptococcales-Tissierellales; f__Peptostreptococcales-Tissierellales;g__Finegoldia	88	Higher in vaginal delivery
DeliveryMethod	CB		–	–
DeliveryMethod	GL	d__Bacteria;p__Firmicutes;c__Bacilli;o__Mycoplasmatales; f__Mycoplasmataceae;g__Ureaplasma	49	Higher in vaginal delivery
DeliveryMethod	M	–	–	–
DeliveryMethod	VD	–	–	–
Epidural^a^	AF	N/A	N/A	N/A
Epidural	CB	–	–	–
Epidural	GL	d__Bacteria;p__Firmicutes;c__Bacilli;o__Mycoplasmatales; f__Mycoplasmataceae;g__Ureaplasma	64	Higher with epidural use
Epidural	M	–	–	–
Epidural	VD	–	–	–
PretermBirth37	AF	–	–	–
PretermBirth37	CB	Unassigned;__;__;__;__;__^b^	27	Lower in preterm birth
PretermBirth37	CB	d__Bacteria;__;__;__;__;__^c^	25	Lower in preterm birth
PretermBirth37	GL	–	–	–
PretermBirth37	M	–	–	–
PretermBirth37	VD	–	–	–
HasGDM	AF	–	–	–
HasGDM	CB	d__Bacteria;p__Actinobacteriota; c__Actinobacteria;o__Frankiales; f__Nakamurellaceae; g__Nakamurella	37	Higher with GDM
HasGDM	GL	–	–	–
HasGDM	M	–	–	–
HasGDM	VD	–	–	–
InducedLabor	AF	–	–	–
InducedLabor	CB	–	–	–
InducedLabor	GL	–	–	–
InducedLabor	M	–	–	–
InducedLabor	VD	–	–	–
IVFET	AF	–	–	–
IVFET	CB	–	–	–
IVFET	GL	–	–	–
IVFET	M	–	–	–
IVFET	VD	–	–	–
Hypertension	AF	d__Bacteria;p__Proteobacteria;c__Gammaproteobacteria;o__Enterobacterales; f__Enterobacteriaceae;g__Escherichia-Shigella	27	Higher with chronic hypertension
Hypertension	CB	d__Bacteria;p__Actinobacteriota;c__Actinobacteria;o__Actinomycetales; f__Actinomycetaceae;g__Actinomyces	87	Higher with preeclampsia
Hypertension	GL	d__Bacteria;p__Actinobacteriota;c__Actinobacteria;o__Bifidobacteriales; f__Bifidobacteriaceae;g__Bifidobacterium	38	Higher with preeclampsia
Hypertension	GL	d__Bacteria;p__Bacteroidota;c__Bacteroidia;o__Bacteroidales; f__Porphyromonadaceae;g__Porphyromonas	29	Higher with preeclampsia
Hypertension	M	d__Bacteria;p__Bacteroidota;c__Bacteroidia;o__Chitinophagales; f__Chitinophagaceae;g__Vibrionimonas	35	Higher with chronic hypertension
Hypertension	VD	d__Bacteria;p__Campilobacterota;c__Campylobacteria;o__Campylobacterales; f__Campylobacteraceae;g__Campylobacter	45	Higher with chronic hypertension
Hypertension	VD	d__Bacteria;p__Firmicutes;c__Clostridia;o__Lachnospirales; f__Lachnospiraceae;g__[Ruminococcus]_torques_group	35	Higher with chronic hypertension
Hypertension	VD	d__Bacteria;p__Actinobacteriota;c__Coriobacteriia;o__Coriobacteriales; f__Coriobacteriaceae;g__Collinsella	34	Higher with chronic hypertension
Hypertension	VD	d__Bacteria;p__Firmicutes;c__Clostridia;o__Peptostreptococcales-Tissierellales; f__Peptostreptococcales-Tissierellales;g__Fenollaria	34	Higher with chronic hypertension
AntibioticsUse	AF	–	–	–
AntibioticsUse	CB	–	–	–
AntibioticsUse	GL	d__Bacteria;p__Bacteroidota;c__Bacteroidia;o__Bacteroidales; f__Tannerellaceae;g__Parabacteroides	40	Higher with antibiotics use
AntibioticsUse	GL	d__Bacteria;p__Proteobacteria;c__Gammaproteobacteria;o__Burkholderiales; f__Burkholderiaceae;g__Cupriavidus	39	Higher with antibiotics use
AntibioticsUse	M	–	–	–
AntibioticsUse^a^	VD	N/A	N/A	N/A
BabySex	AF	–	–	–
BabySex	CB	–	–	–
BabySex	GL	–	–	–
BabySex	M	–	–	–
BabySex	VD	–	–	–

AF, amniotic fluid; CB, umbilical cord blood; GL, gastric liquid; M, meconium; VD, cervicovaginal discharge.

^a^Significant hits were found by ANCOM, but these results were discarded as they have a very low W score (zero in many cases) and are likely artifacts; note that this is a known bug in ANCOM, typically caused by small sample size for a given test.

^b^Amplicon sequence variants were labelled ‘Unassigned’ if it was not possible to classify them at the highest taxonomic level at the required confidence level.

^c^These amplicon sequence variants could not be classified beyond the domain level at the required confidence level.

### The resemblance of twin microbiome in delivery

**Figure 4:**
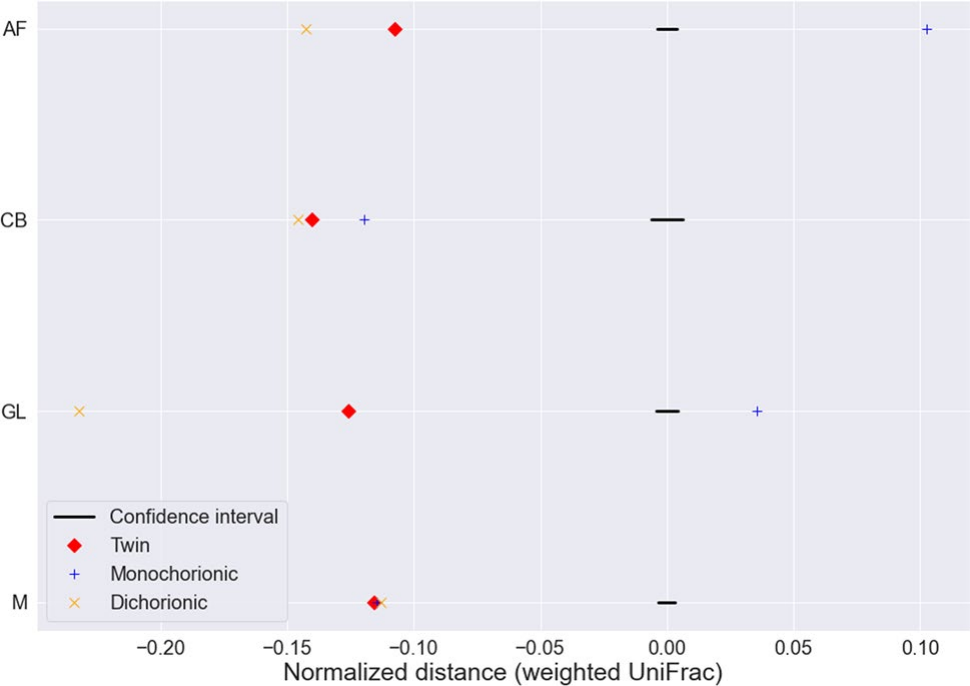
Higher similarity of microbiome composition in twin samples than in randomly chosen samples. For each sample type, the means of weighted UniFrac distances are shown for the twin samples. A 95% confidence interval was constructed by randomly sampling pairwise distances with replacement from the samples for 1000 times.

To test the hypothesis that samples from twins, both monochorionic and dichorionic, have higher similarity in microbiome composition than randomly chosen samples, we compared the mean of weighted UniFrac distance between twin samples and randomly selected samples. More specifically, for each of the AF, CB, GL, and M groups, we performed bootstrapping hypothesis testing by randomly sampling pairwise distances with replacement from all samples 1000 times to build a 95% confidence interval with the means of the sampled distances. We rejected the null hypothesis that there was no difference between the twin samples and randomly selected samples for all four sample types because the mean pairwise distance for twin samples was below the confidence interval (Fig. 4 and Supplementary Fig. S7). Next, we divided the twins into monochorionic and dichorionic twins and repeated hypothesis testing. We found that we could still reject the null hypothesis for all four sample types for dichorionic twins. For monochorionic twins, however, only the CB and M groups passed the test.

### Characterization of the vaginal health-related microbiome

**Figure 5:**
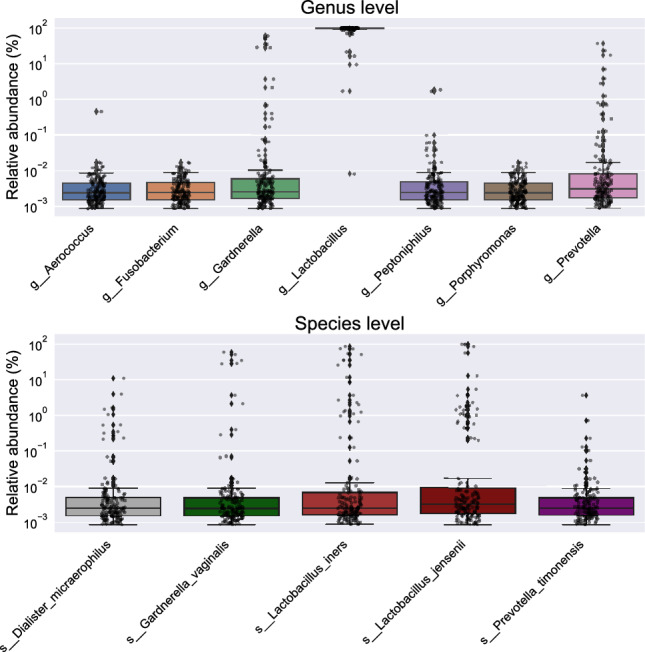
Relative abundance of bacteria associated with vaginal health. Only bacterial targets in uBiome’s SmartJane assay that are also present in the vaginal discharge samples are shown.

Several pathogenic and commensal vaginal microbiota have been shown to have important consequences for a woman’s reproductive and general health. To establish reference ranges of vaginal microbiota with known clinical associations in generally healthy pregnant women, we searched for bacterial targets commonly tested for assessing vaginal health within VD samples. More specifically, we focused on 31 bacterial targets (15 genera and 16 species) that are tested by the “SmartJane” assay from uBiome Inc., including *Lactobacillus*, *Sneathia*, and *Gardnerella*^21^. Of the 31 bacterial taxa of clinical importance, 12 were identified in our samples (Fig. 5).

We observed a higher relative abundance of *Lactobacillus* at the genus level but lower abundances of *Aerococcus*, *Fusobacterium*, *Gardnerella*, *Peptoniphilus*, *Porphyromonas*, and *Prevotella*. Most of our patients did not have any severe pregnancy-related complications. In addition, the majority of preterm birth ranged in the late preterm period from 34 + 0 weeks to 36 + 6 weeks. Therefore, the “SmartJane” assay did not capture almost any pathogenic microbiome. The specification level was examined and is listed in Fig. 5. We found *Lactobacillus iners* and *Lactobacillus jensenii* from the assay lists, but *Lactobacillus crispatus* was not commonly found in the vaginal microbiome. This could be simply because the SILVA reference database we used omitted *Lactobacillus crispatus*. We confirmed that some of the ASVs from the *Lactobacillus* genus were indeed *Lactobacillus crispatus* using the National Center for Biotechnology Information (NCBI) database (data not shown).

## Discussion

### Controversies surrounding in utero colonization

Since contamination is a critical issue in microbiome research, we used several up-to-date methods to confirm the presence of bacteria and found evidence of in utero colonization. The distinct bacterial composition in meconium, the first stool passed by newborns, supports that microbial colonization occurs in the intrauterine environment during normal pregnancy^22,23^. The microbiome in neonatal gastric liquid was similar to that in maternal amnionic fluid, as expected since fetuses swallow amnionic fluid in utero and their urine returns to the fluid under normal physiological conditions. However, the microbiome in gastric liquid was not exactly the same as in amnionic fluid, indicating the existence of unknown mechanisms for flora formation in the fetal oral cavity or proximal gastrointestinal tract, such as esophagus, from the intrauterine environment.

### Do different samples from mothers and newborns share the same microbiome?

The study aimed to determine whether samples from various body sites of pregnant women and their infants would have similar microbiomes, or if the maternal microbiome would be passed on to her fetus. Our results suggest that the microbiome primarily differed based on the body compartment where it was obtained, not the mother-fetus pair. That is, out of all factors, including various obstetric conditions, the sampling site was the most significant factor in determining microbiome similarity.

### Establishing a representative microbiome library of various samples to understand the microbiomes of typical pregnant women, fetuses, and neonates

This study’s key strength is its study population, which comprised of pure Asians and reflected general, low-risk pregnancies. The maternal age range was between 20 and 45 years, which is considered typical for reproductive age. There were roughly equal numbers of nulliparous women, caesarean sections, and male and female neonates. Other than a small number of instances of fetal distress, such as low Apgar scores and meconium staining, newborns with extremely pathological conditions that could alter the microbiome, such as severe preterm birth and treatment in a neonatal intensive care unit (NICU), were excluded. As a result, the microbiome analyzed in this study population is likely to represent typical pregnancy. It is crucial to establish a microbiome library for low-risk pregnant women and their normal neonates as a basis for comparison with pathological conditions, to better understand the microbiome composition during pregnancy.

### Association between microbiome and various pregnancy-related phenotypes

To identify the microbiomes associated with pregnancy-related conditions, such as delivery method, we conducted statistical analysis of differential abundance. Despite the challenges posed by the low microbial biomass and difficulties in controlling study subjects, which can result in false positive results, the bacteria listed in Table 3 seem to align with previous findings. For example, *Finegoldia* and *Bifidobacterium* have been previously linked to a healthier pregnancy, and our data confirms this association^24,25^. Other taxa listed in the table also have links to inflammation and pregnancy complications, such as gestational diabetes mellitus, preeclampsia, and preterm birth. The presence of *Campylobacter* and *Lachnospiraceae* in vaginal discharge, for example, is in line with previous research showing that these bacterial infections can lead to inflammation and preterm birth^26,27^.

By cross-referencing with clinical databases, our analysis revealed several significant associations. First, the abundant presence of *Lactobacillus* and *Gardnerella* in vaginal discharge is a well-known indicator of the pregnancy microbiome. *Lactobacillus* plays a protective role in the maternal microbiome during pregnancy, while *Gardnerella* is considered a pathogen and is strongly associated with preterm birth or pregnancy complications^11,13,26^. The presence of *Faecalibacterium* in cord blood is noteworthy, as it has been shown to be depleted in gestational diabetes mellitus^28^, even though the number of cases in our study population was relatively small. Additionally, *Staphylococcus* was found to be strongly associated with preterm birth in meconium. This result coincides with previous findings that suggest *Staphylococcus* infections can lead to preterm birth^29,30^.

Regarding the effect of antibiotics, we analyzed the relationship between antibiotic use and meconium samples, but the results showed limited association due to the small sample size. As Tormo-Badia et al. reported, antibiotics can alter the gut microbiome of offspring in pregnant mice^31^. Given that the existence of a “healthy microbiome” during pregnancy is considered crucial for maintaining a normal pregnancy, it is easy to imagine the potential negative consequences of antibiotics administration during pregnancy. Since antibiotics are only given to pregnant women who have signs of infection or inflammation, specific diseases, or preterm premature rupture of membranes with the risk of ascending infection to the fetus, it is practically challenging to determine the effect of antibiotics on the modification of the birth-related microbiome.

The meconium samples showed the presence of microbiome taxa such as *Lactobacillus*, *Staphylococcus*, and *Ureaplasma*, which are collectively known as the vaginal flora^11,32^. We attempted to evaluate the relationship between delivery mode and the microbiome in meconium, but we did not find any statistically significant differences in composition or diversity. According to a study by Dominguez-Bello et al., there are differences in the bacterial communities in the guts of infants depending on the mode of delivery^33^. Neonates born vaginally have a microbiome resembling their mother's vaginal microbiota, dominated by *Lactobacillus*. Conversely, infants born via cesarean section have a microbiome dominated by *Staphylococcus*, *Corynebacterium*, and *Propionibacterium*, which are commonly found on their mothers’ skin surfaces.

### Twin pregnancy and microbiome

Approximately a quarter of the pregnancies in our study were twin pregnancies (37/141). To the best of our knowledge, this is the first study to assess the microbiomes of twin newborns. Generally, our twin samples (AF, CB, GL, and M) showed a more similar composition compared to randomly selected samples, even for dichorionic twins who have separate intrauterine compartments. The only exception was CB and M samples from monochorionic twins, where randomly selected samples showed greater similarity, which is likely due to the small sample size of monochorionic twins.

## Conclusion

Exploring the microbiologic features related to pregnancy has been a challenging and controversial task for many years. Microbial invasion of the gestational cavity such as amniotic fluid or placenta can lead to serious obstetric complications such as preterm birth and severe neonatal morbidities that may persist throughout life. Despite the importance of research on the microbiome in pregnancy, progress has been limited due to ethical and accessibility issues. We have collected various samples from pregnant women and their neonates using a standardized protocol and established a microbiome database, which can serve as a reference library for studying samples with other pregnancy-related or pathologic conditions.

## Methods

### Study design and sample collection

A prospective study was performed on live births delivered between March 2020 and January 2021. Samples were collected from women who had delivered at Seoul National University Bundang Hospital and their newborns. Women with unstable vital signs or those requiring urgent management such as transfusion and neonates admitted to the NICU or who had unstable vital signs after birth were excluded from the study. Samples for microbiome analysis included maternal VD, AF, CB, neonatal GL, and M. As a pregnant woman was hospitalized with expectancy of delivery, the VD sample was obtained using a polyester swab inserted into the posterior fornix of the vagina, assisted by sterile speculum examination. For those who had undergone cesarean section for delivery or amniocentesis for specific indications (i.e., for detection of intraamniotic inflammation/infection), approximately 10 cc of AF was obtained through a syringe for the study. During delivery, both cesarean section and vaginal delivery, approximately 20 cc of CB was taken through a syringe from the vein of the umbilical cord immediately after clamping. The syringe needle was directly inserted into the umbilical cord at the delivery site surrounded by sterile drapes to minimize surgical field contamination. Since removing amniotic fluid or other liquid from the newborn’s mouth and stomach after birth is a part of initial management to help the airway and to stimulate spontaneous breathing, most neonates received suctioning procedures, and the liquid collected in the suction bottle (approximately 15 ml) was carried into a conical tube for analysis of GL. The M sample, the newborn's very early stool, was carefully obtained within 24 h after birth using a polyester swab inserted into the anus as the neonate stabilized after initial management. We tried to collect all five different samples from each woman and neonate(s), nonetheless, a small part of samples from mother-neonate pairs were not obtained or missed for clinical circumstances. The primary outcome was the distribution and composition of the microbiome of the above samples from pregnant women and their neonates. To determine the association between the microbiome from different compartments and obstetric factors, medical records were collected and thoroughly reviewed. Data included maternal age, gestational age at delivery, delivery mode (vaginal delivery or cesarean section), the use of ART, other obstetric complications, and neonatal outcomes such as sex and birth weight.

### Ethics approval and consent to participate

This study was performed with the informed consent of appropriate participants in compliance with the Declaration of Helsinki. The study protocol was approved by the Institutional Review Board of the Seoul National University Bundang Hospital (B-1606/350-003).

### Microbial DNA isolation

Microbial deoxyribonucleic acid (DNA) was extracted from the VD, GL, AF, and CB samples with the ZymoBIOMICS DNA Miniprep Kit (Zymo Research, Irvine, CA) and the sample M using the DNeasy PowerSoil Pro Kit (Qiagen, Germantown, MD) according to the manufacturer’s instructions. Briefly, samples were enzymatically and mechanically lysed by bead beating, followed by washing and filtering in the provided column. Extracted DNA concentrations were measured using a Qubit dsDNA HS Assay Kit (Thermo Fisher Scientific, Waltham, MA, USA). The total amounts of extracted DNA were varied based on sample types, such as 1–10 ug for VD, 3 μg for CB, 30–200 ng for M, and 50 ng for GL and AF. For each box of the DNA extraction kit used, no material was used as a negative control. The blanks were processed in the entire protocol and analyzed.

### 16S rRNA gene amplification

The 16S ribosomal ribonucleic acid (rRNA) gene was amplified using the two-step polymerase chain reaction (PCR) protocol in the 16S Metagenomic Sequencing Library Preparation (Illumina, San Diego, CA). In the first PCR step, the V3–V4 hypervariable region of the 16S rRNA gene was amplified using 10 ng of each sample, 10 µM of 341F/785R primers, and Herculase II fusion DNA polymerase (Agilent, Santa Clara, CA). In the below primer sequence, ‘N’ base is selected from any random base, ‘W’ base is A or T, ‘H’ base is A, C or T, and ‘V’ base is A, C, or G.

341F: 5′- TCGTCGGCAGCGTCAGATGTGTATAAGAGACAGCCTACGGGNGGCWGCAG-3′

785R: 5′-GTCTCGTGGGCTCGGAGATGTGTATAAGAGACAGGACTACHVGGGTATCTAATCC-3′

PCR cycling was performed with an initial cycle at 95 °C for 3 min, followed by 25 cycles of 95 °C for 30 s, 55 °C for 30 s, 72 °C for 30 s, and a final extension cycle at 72 °C for 5 min. The amplicons were cleaned with AMPure XP beads (Beckman Coulter, Brea, CA, USA). In the second PCR, index primers from the Nextera DNA CD Index Kit (Illumina, San Diego, CA) were added to the ends of the amplicons generated in the first PCR. PCR cycling was performed with an initial cycle at 95 °C for 3 min, followed by ten cycles of 95 °C for 30 s, 55 °C for 30 s, 72 °C for 30 s, and a final extension cycle at 72 °C for 5 min. Each sample was cleaned with AMPure XP beads (Beckman Coulter, Brea, CA, USA) and eluted in UltraPure DNase/RNase-Free Water (Thermo Fisher Scientific, Waltham, MA). The amplified DNA was checked using a 2100 Bioanalyzer system using an Agilent DNA 1000 Kit (Agilent, Santa Clara, CA, USA). For each library production, no template was used as a negative control.

### 16S rRNA gene sequencing and analysis

Based on the DNA size and concentration, the amplicons were pooled in equimolar amounts and spiked with 30% PhiX (Illumina, San Diego, CA). These were then sequenced on the Illumina MiSeq platform using paired-end 250 cycle MiSeq Reagent Kit V2 (Illumina, San Diego, CA) and a 300 cycle MiSeq Reagent Kit V3 (Illumina, San Diego, CA). Negative controls from the DNA extraction and library were sequenced.

### Sequencing data generation

We divided the samples into nine batches (Runs 1–9) and sequenced the V3-V4 region of the 16S rRNA gene using Illumina MiSeq machines with a target depth of 100,000 per sample (Supplementary Fig. S8). Sequencing was performed with 250 bp paired-end reads for all of the sequencing runs except for the last one (Run 9), where sequencing was performed with 300 bp paired-end reads for practical reasons. The read quality scores for each sequencing run are shown in Supplementary Fig. S9. The bcf2fastq program of Illumina was used to demultiplex raw sequencing data (BCL files) and output forward and reverse FASTQ files for each sample. Of note, some samples were sequenced more than once to assess the impact of batch effects. These included “sequencing duplicates” in which the identical NGS library of one sample was sequenced in separate runs and “library duplicates” in which multiple NGS libraries were prepared from the identical sample at different dates and then sequenced separately.

### Data analysis and visualization

Unless stated otherwise, all analyses were carried out using the QIIME 2 platform, a powerful community-developed platform for microbiome bioinformatics^34^. For each sequencing run, FASTQ files were imported to QIIME 2 and the DADA2 plugin^35^ to identify ASVs by trimming low-quality parts of sequence reads, denoising trimmed reads, and then merging the forward and reverse reads (Supplementary Fig. S8). The observed ASVs from individual sequencing runs were then merged into one ASV table. To detect and remove potential contaminants, we ran the decontam program on our samples, which looked for ASVs per sequencing batch that appeared at higher frequencies in low-concentration samples and were repeatedly found in the negative control^36^. Taxonomy classification was performed using a naive Bayes classifier using the SILVA database^37^. To visualize the outputs from QIIME 2, we developed the Dokdo program (https://github.com/sbslee/dokdo), an open-source and MIT-licensed Python package for microbiome sequencing analysis using QIIME 2. Dokdo internally uses the application programming interface of QIIME 2 and therefore does not require any other dependencies. Dokdo can be used to perform a variety of secondary analyses or create publication-quality figures from QIIME 2 files/objects (e.g. a taxonomic bar plot or an alpha rarefaction plot).

### Diversity analysis

We used the QIIME 2 command “qiime diversity core-metrics-phylogenetic” to compute the alpha and beta diversity metrics of our samples. When running the command, to normalize for the difference in read depth across the samples, we used the “-p-sampling-depth” option to rarefy our samples to 5,000 sequence reads and have an equal depth of coverage. We also ensured that all samples were sequenced to a sufficient depth of coverage for diversity analysis by creating rarefaction curves (Supplementary Fig. S10). Additionally, we used the “-i-phylogeny” option to provide a rooted phylogenetic tree of observed ASVs, which is required for performing PCoA based on the weighted UniFrac distance^38^.

### Statistical analysis

To assess the differential abundance of the microbiome in the context of clinical information such as preterm birth, we used the QIIME 2 command “qiime composition ancom” to perform ANCOM, which compares the centered log-ratio (CLR) of relative abundance between two or more groups of samples^39^. To determine whether groups of samples are significantly different from one another in beta diversity, we carried out PERMANOVA using the QIIME 2 command “qiime diversity adonis” which fits linear model assumptions to a distance matrix (e.g., weighted UniFrac) with the chosen variables. We performed bootstrapping hypothesis testing by building a 95% confidence interval with the “scipy.stats.t.interval” method in the scipy package to compare similarities in microbiome composition between twins and randomly chosen samples^40^.

### Supplementary Information

Supplementary Figures.

## Data Availability

The sequencing data generated from this study has been deposited in the INSDC databases through the European Nucleotide Archive (ENA) under accession number PRJEB52455. The ENA URL is https://www.ebi.ac.uk/ena/browser/view/PRJEB52455. The data generated during the current study are available from the corresponding author on reasonable written request.

## Abbreviations

AF: Amniotic fluid
ANCOM: Analysis of the composition of microbiomes
ART: Assisted reproductive technology
ASVs: Amplicon sequence variants
BMI: Body mass index
CB: Umbilical cord blood
CLR: Centered log-ratio
DNA: Deoxyribonucleic acid
GL: Gastric liquid
IUI: Intrauterine insemination
IVF-ET: In vitro fertilization and embryo transfer
M: Meconium
NCBI: National Center for Biotechnology Information
NGS: Next-generation sequencing
NICU: Neonatal intensive care unit
PCoA: Principal coordinates analysis
PCR: Polymerase chain reaction
PERMANOVA: Permutational multivariate analysis of variance
RNA: Ribosomal ribonucleic acid
VD: Cervicovaginal discharge
