# Author Correction: Comprehensive characterization of maternal, fetal, and neonatal microbiomes supports prenatal colonization of the gastrointestinal tract

**DOI:** 10.1038/s41598-023-46309-3

**Published:** 2023-11-02

**Authors:** Jee Yoon Park, Huiyoung Yun, Seung-been Lee, Hyeon Ji Kim, Young Hwa Jung, Chang Won Choi, Jong-Yeon Shin, Joong Shin Park, Jeong-Sun Seo

**Affiliations:** 1 Department of Obstetrics and Gynecology, Seoul National University College of Medicine, Seoul, Republic of Korea; 2 Department of Obstetrics and Gynecology, Seoul National University Bundang Hospital, Gyeonggi-do, Republic of Korea; 3 Department of Pediatrics, Seoul National University College of Medicine, Seoul, Republic of Korea; 4 Department of Pediatrics, Seoul National University Bundang Hospital, Gyeonggi-do, Republic of Korea; 5 Precision Medicine Center, Seoul National University Bundang Hospital, Gyeonggi-do, Republic of Korea; 6 Macrogen Inc, Seoul, Republic of Korea

Correction to: *Scientific Reports* 10.1038/s41598-023-31049-1, published online 21 March 2023

The original version of this Article contained an error in the Acknowledgements section.

“This study was supported by Seoul National University Bundang Hospital Research Fund (14-2017-026) and World-class 300 project by Ministry of SMEs and Start-ups.”

now reads

“This study was supported by Seoul National University Bundang Hospital Research Fund (18-2018-002) and World-class 300 project by Ministry of SMEs and Start-ups.”

The original Article has been corrected.

