# Supplementary material for: Comprehensive characterization of maternal, fetal, and neonatal microbiomes supports prenatal colonization of the gastrointestinal tract
Source: Sci Rep. 2023 Mar 21;13:4652. doi: 10.1038/s41598-023-31049-1 (PMC10030461; doi:10.1038/s41598-023-31049-1)
Supplement: Supplementary file 1 — Supplementary Figures. [file 41598_2023_31049_MOESM1_ESM.pdf]

Comprehensive Characterization of Maternal, Fetal, and Neonatal Microbiome

Jee Yoon Park[1,2#], Huiyoung Yun[4,5#], Seung-been Lee[4,5], Hyeon Ji Kim[2], Young Hwa Jung[3], Chang Won Choi[3], Jong-Yeon Shin[4,5], Joong Shin Park[1*], Jeong-Sun Seo[4,5*]

[1] Department of Obstetrics and Gynecology, Seoul National University College of Medicine, Seoul, Republic of Korea

[2] Department of Obstetrics and Gynecology, Seoul National University Bundang Hospital, Gyeonggi-do, Republic of Korea

[3] Department of Pediatrics, Seoul National University Bundang Hospital, Gyeonggi-do, Republic of Korea

[4] Precision Medicine Center, Seoul National University Bundang Hospital, Gyeonggi-do, Republic of Korea

[5] Macrogen Inc, Seoul, Republic of Korea

[#] Theses authors contributed equally to the work

***Corresponding authors:** jsparkmd@snu.ac.kr; jeongsun@snu.ac.kr

Supplementary Figures

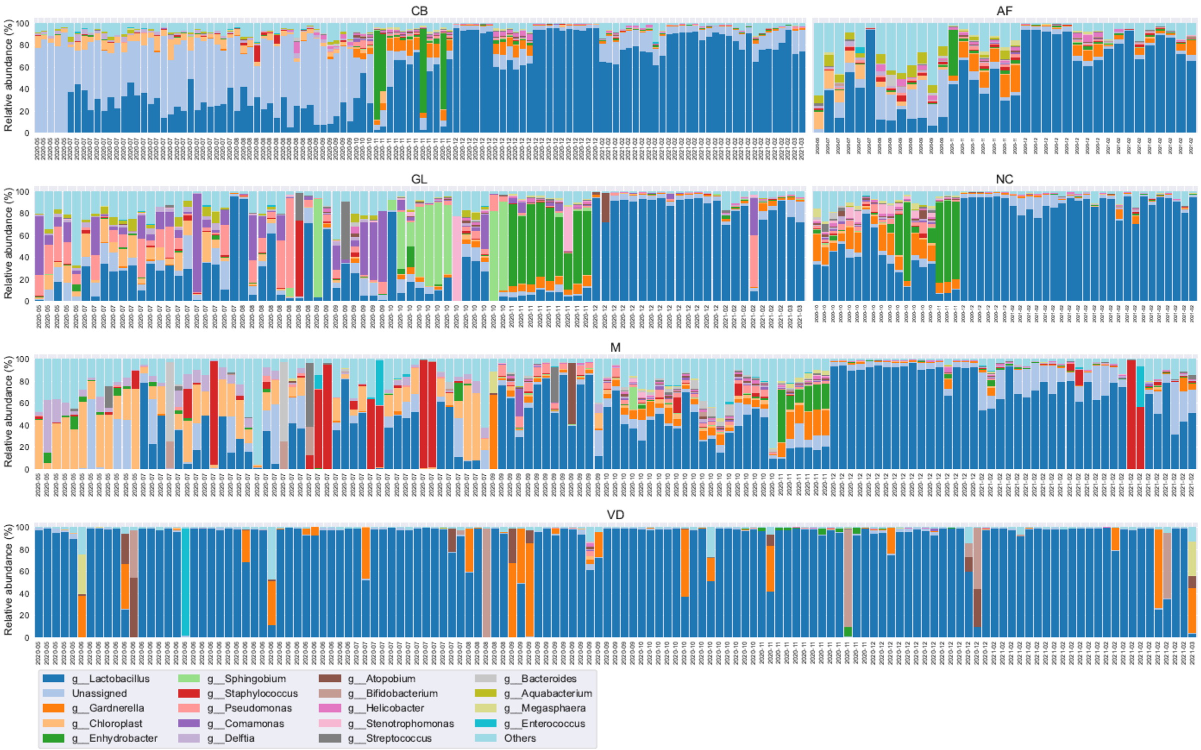

Supplementary Figure S1. Relative microbiome abundance at the genus level without removal of contaminants. The 19 most abundant genera are labelled. Abbreviations: AF, amniotic fluid; CB, cord blood; GL, gastric liquid; NC, negative control; M, meconium; VD, vaginal discharge.

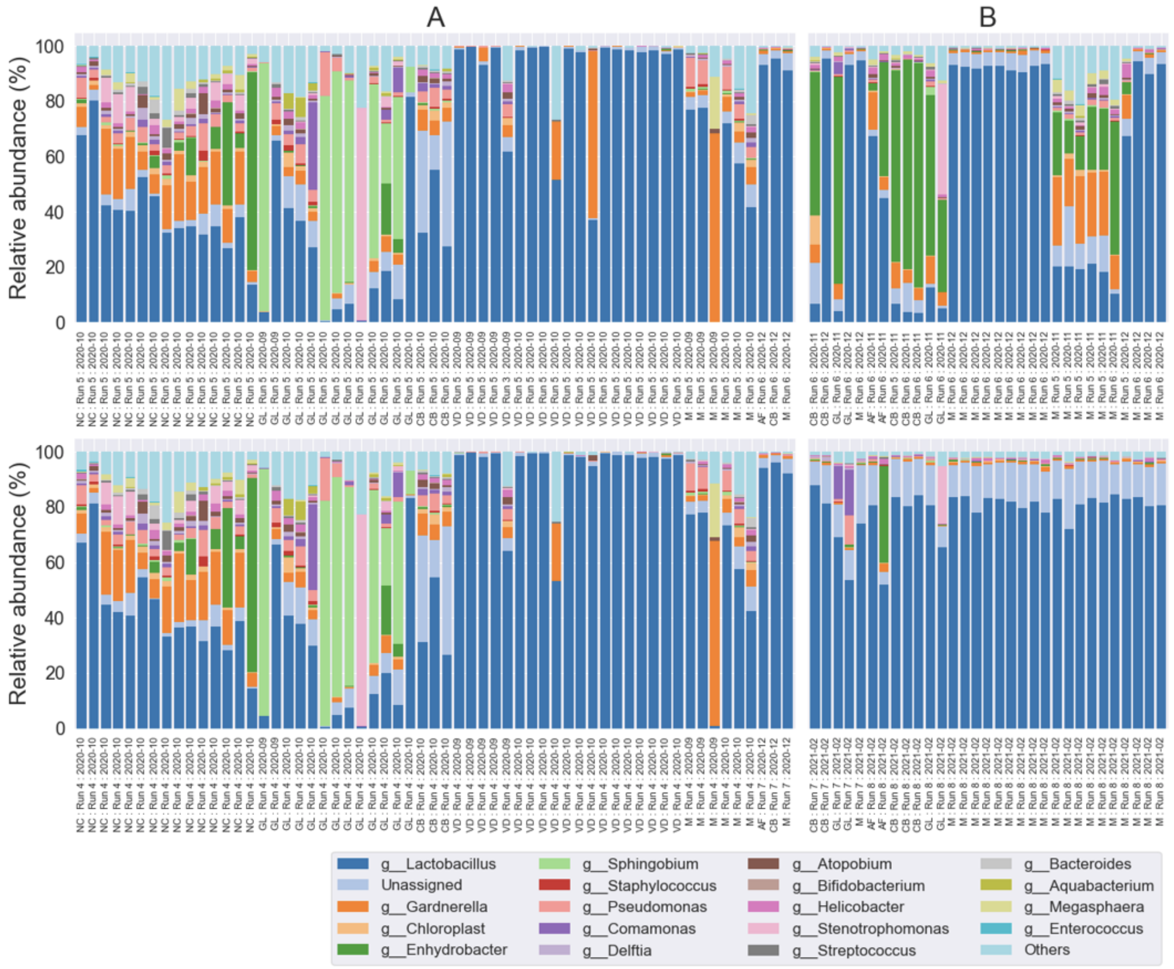

Supplementary Figure S2. Microbiome characterization of the sequencing or library replicates. Bar charts of unfiltered microbiome at the genus level are shown for (A) the sequencing replicates and (B) the library replicates. The upper row indicates samples included in the main analyses while the lower row indicates corresponding replicates. The 19 most abundant genera are labelled. Abbreviations: AF, amniotic fluid; CB, cord blood; GL, gastric liquid; NC, negative control; M, meconium; VD, vaginal discharge.

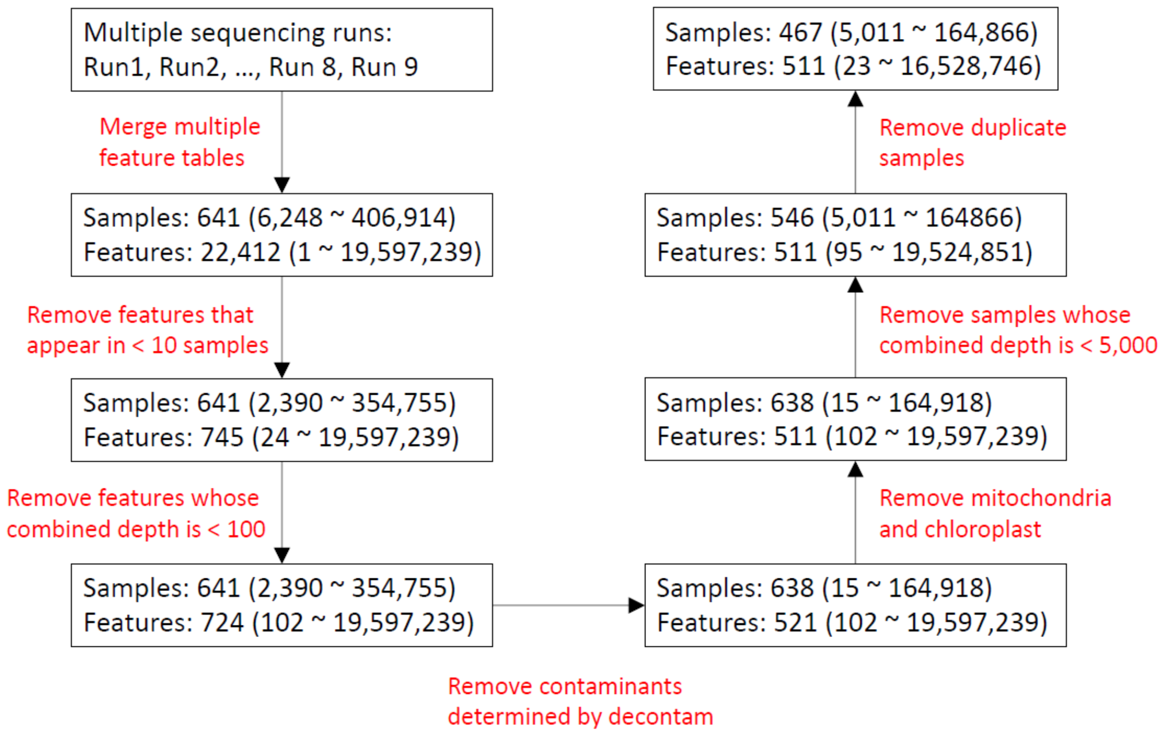

Supplementary Figure S3. Filtering scheme for contaminant removal.

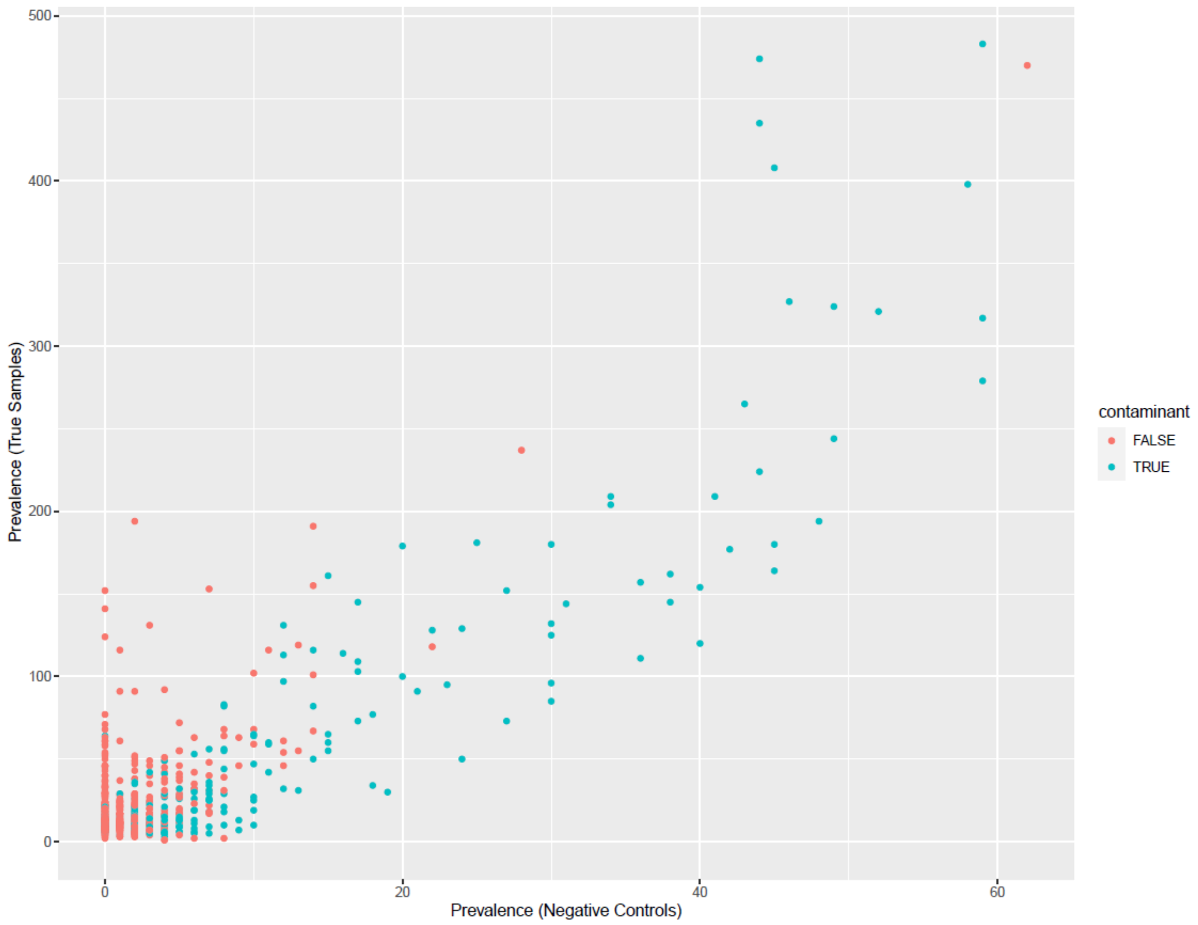

Supplementary Figure S4. Prevalence of contaminants in true samples vs. negative controls determined by the decontam program.

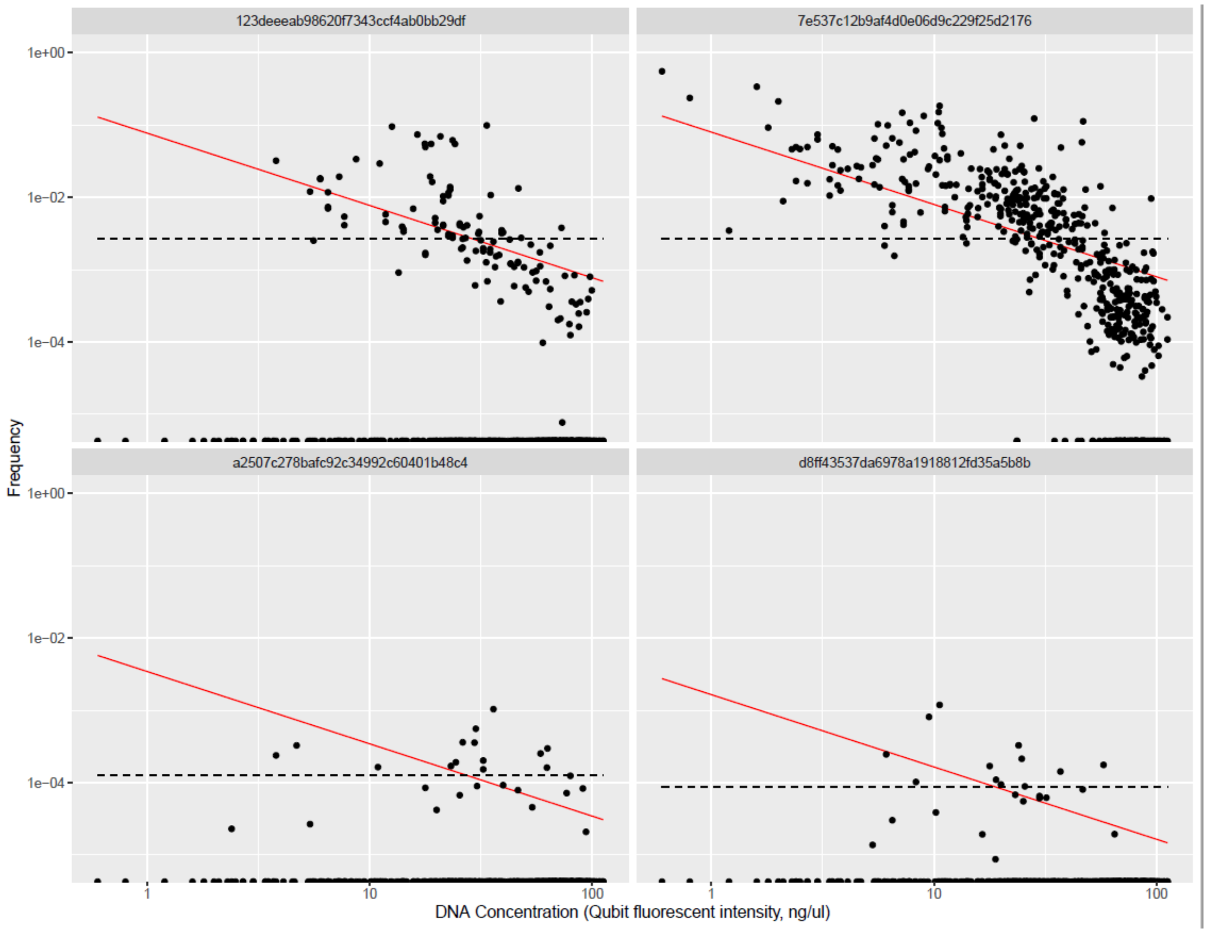

Supplementary Figure S5. Examples of contaminants and their abundance determined by the decontam program.

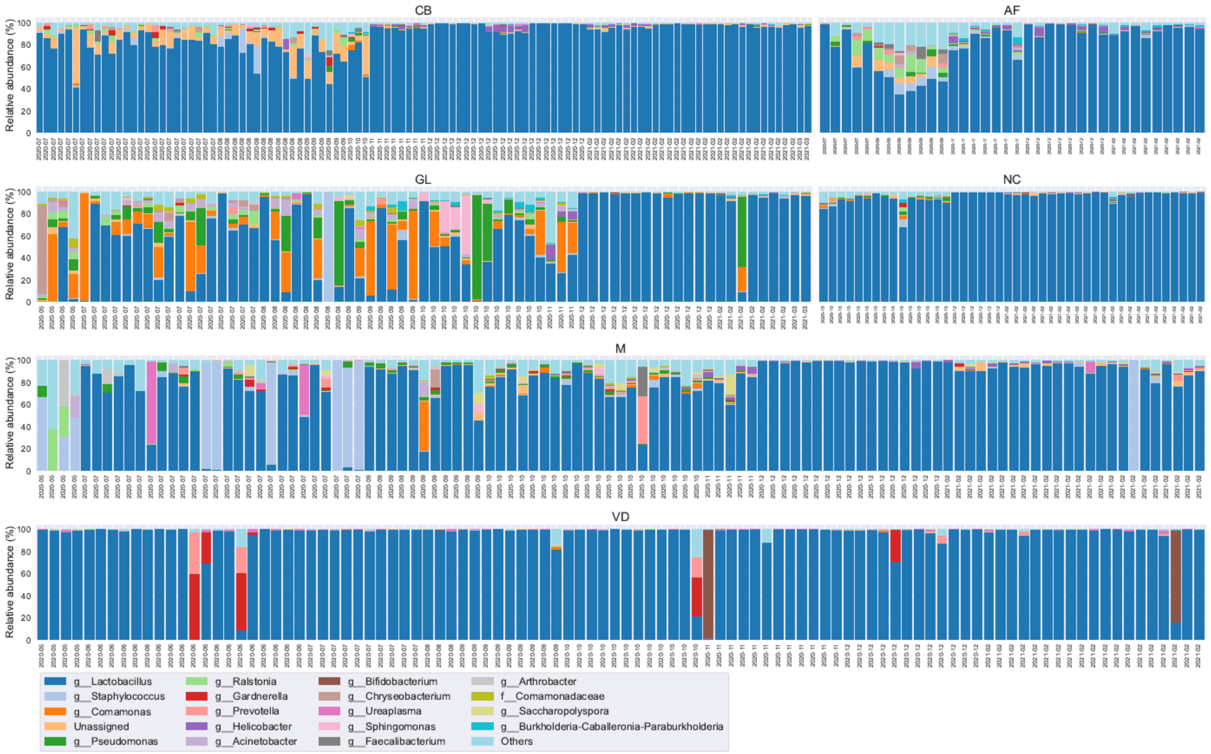

Supplementary Figure S6. Relative microbiome abundance at the genus level after removal of contaminants. The 19 most abundant genera are labelled. Abbreviations: AF, amniotic fluid; CB, cord blood; GL, gastric liquid; NC, negative control; M, meconium; VD, vaginal discharge.

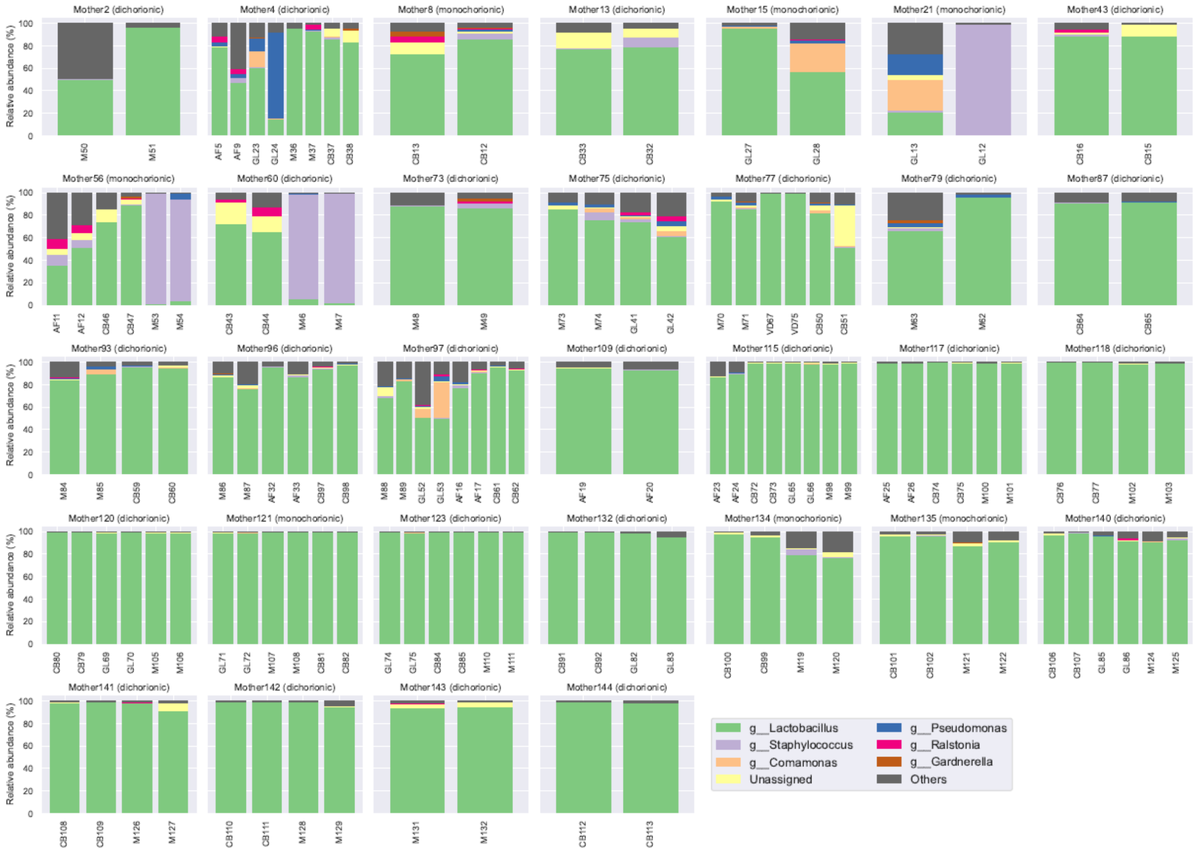

Supplementary Figure S7. Microbiome characterization of the twin samples. Bar charts of unfiltered microbiome at the genus level are shown. The six most abundant genera are labelled. Abbreviations: AF, amniotic fluid; CB, cord blood; GL, gastric liquid; NC, negative control; M, meconium; VD, vaginal discharge.

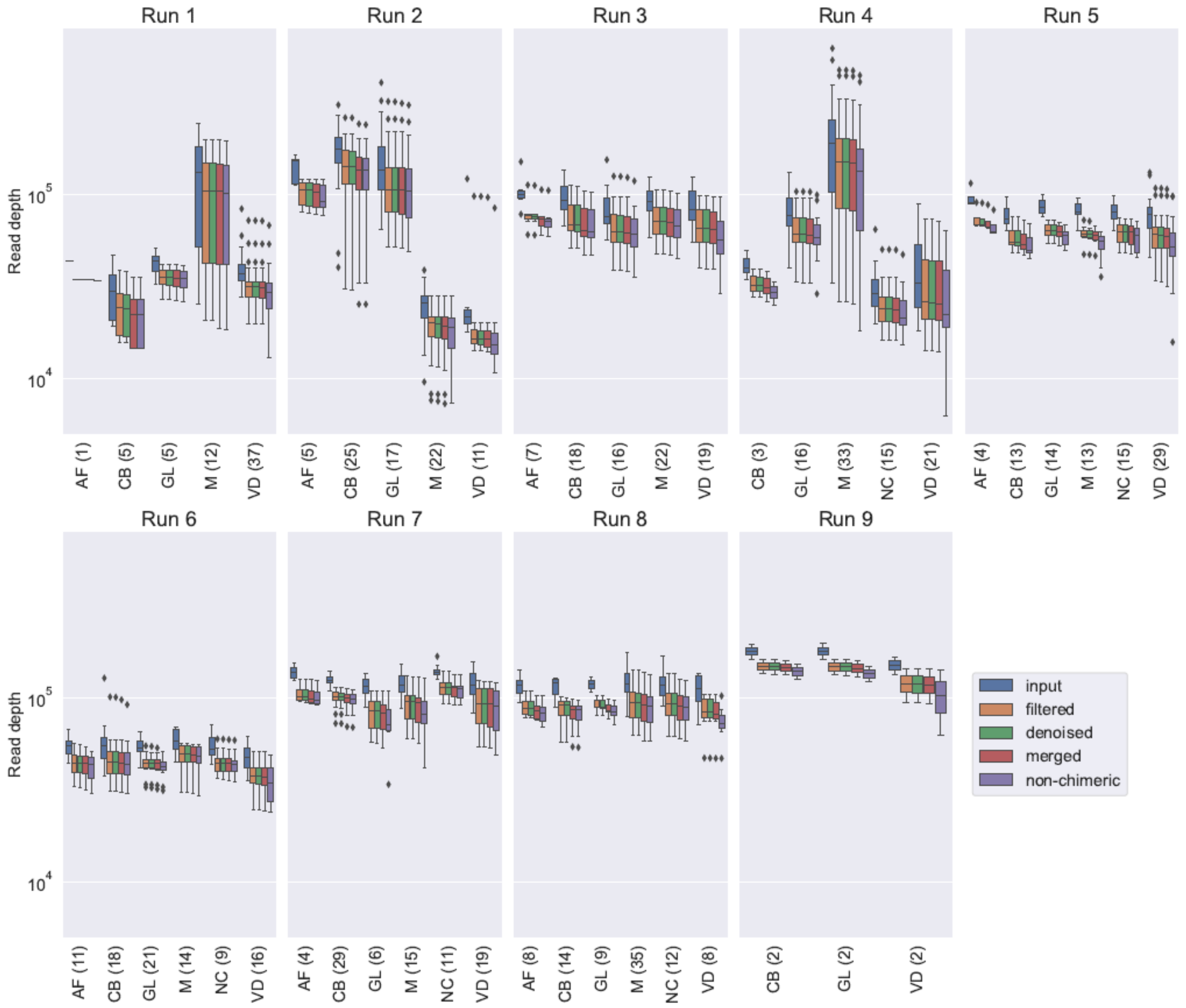

Supplementary Figure S8. Denoising results from the DADA2 program. Abbreviations: AF, amniotic fluid; CB, cord blood; GL, gastric liquid; NC, negative control; M, meconium; VD, vaginal discharge.

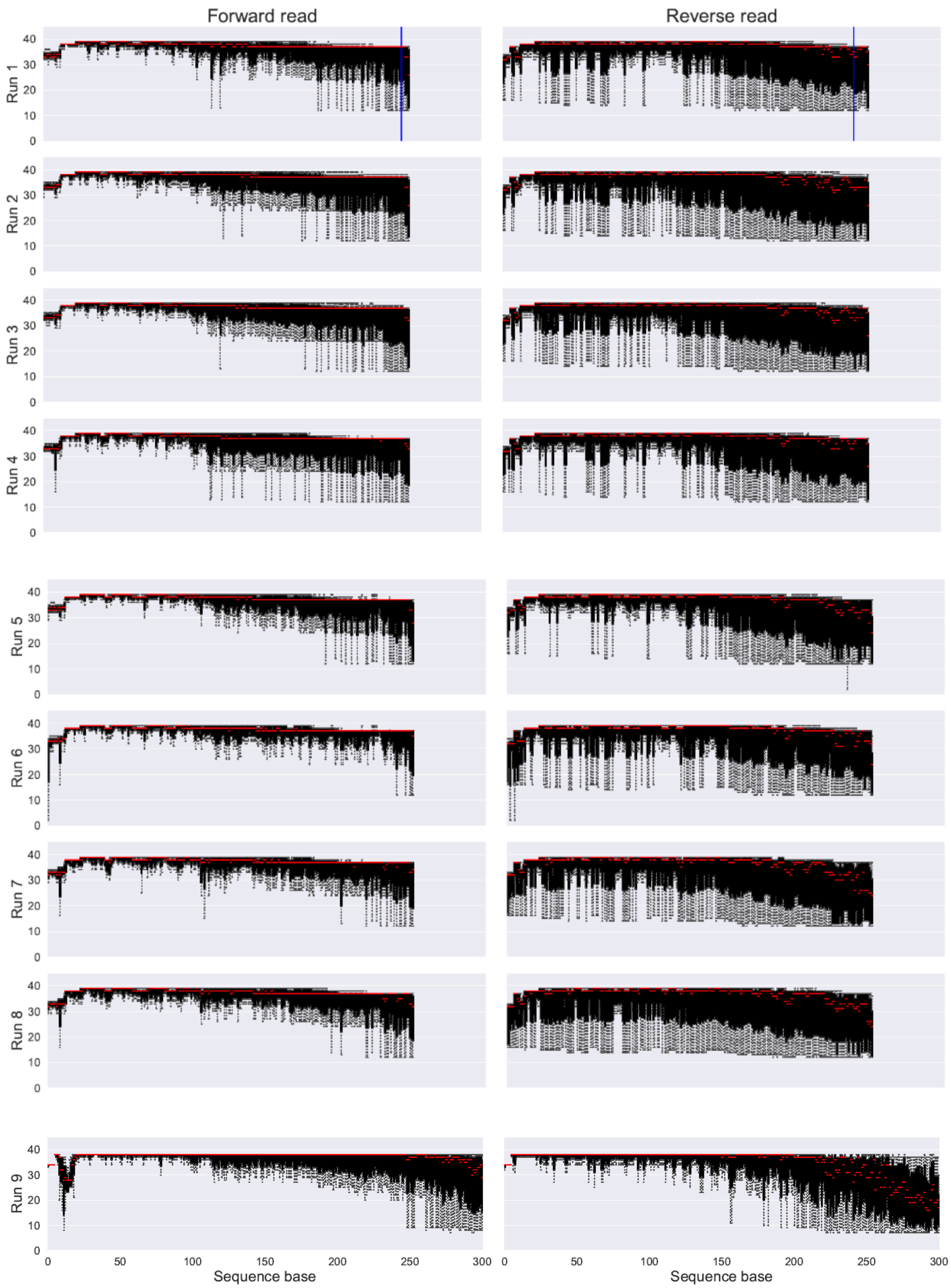

Supplementary Figure S9. Assessment of read quality. Sequencing was performed using 250 bp paired-end reads for all runs except for Run 9 in which 300 bp was used instead due to

practical reasons. Forward and reverse reads were truncated to 245 bp and 240 bp (indicated in blue vertical line), respectively, before they were denoised by the DADA2 program.

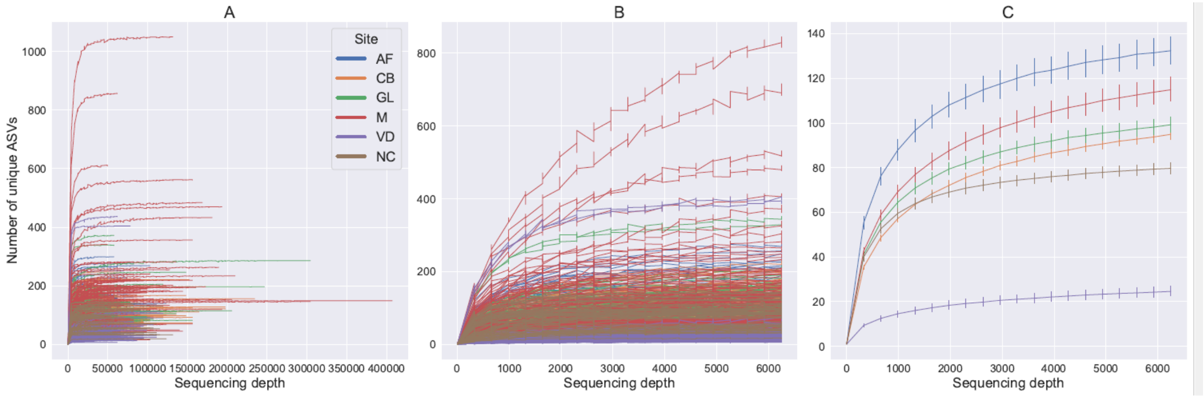

Supplementary Figure S10. Rarefaction curves of the study samples. (A) Sequence reads of each sample were sequentially down-sampled to generate rarefaction curves. (B) Same as (A), but zoomed in on the smaller depth. (C) Same as (B), but with the samples aggregated by origin. Abbreviations: AF, amniotic fluid; CB, cord blood; GL, gastric liquid; NC, negative control; M, meconium; VD, vaginal discharge.
